# Integrated MXene and metal oxide electrocatalysts for the oxygen evolution reaction: synthesis, mechanisms, and advances

**DOI:** 10.1039/d4sc04141k

**Published:** 2024-08-26

**Authors:** Muhammad Nazim Lakhan, Abdul Hanan, Yuan Wang, Hiang Kwee Lee, Hamidreza Arandiyan

**Affiliations:** a Applied Chemistry and Environmental Science, School of Science, STEM College, RMIT University Melbourne VIC 3000 Australia; b Sunway Centre for Electrochemical Energy and Sustainable Technology (SCEEST), School of Engineering and Technology, Sunway University Selangor 47500 Malaysia; c Department of Chemical Engineering, The University of Melbourne Parkville VIC 3010 Australia; d School of Chemistry, Chemical Engineering and Biotechnology, Nanyang Technological University 21 Nanyang Link Singapore 637371 Singapore; e Centre for Applied Materials and Industrial Chemistry (CAMIC), School of Science, RMIT University Melbourne VIC 3000 Australia hamid.arandiyan@rmit.edu.au

## Abstract

Electrochemical water splitting is a promising approach to produce H_2_ through renewable electricity, but its energy efficiency is severely constrained by the kinetically slow anodic oxygen evolution reaction (OER), which uses about 90% of the electricity in the water-splitting process due to its multistep proton (H^+^)-coupled electron (e^−^) transfer process, high overpotential (*η*), and low energy efficiency. Therefore, the quest for efficient, sustainable, and cost-effective electrocatalysts for hydrogen production through water electrolysis has intensified, highlighting the potential of two-dimensional (2D) MXenes. MXenes have emerged as a promising class of materials characterized by excellent stability, hydrophilicity, and conductivity. However, challenges such as low oxidation resistance, facile stacking, and the absence of intrinsic catalytically active sites limit their performance. This review thoroughly explores various synthesis methods for MXenes and their integration with transition metal oxides (TMOs) to tackle the challenges and enhance catalytic activity. The review also delves into advanced strategies for structural tuning of MXenes and TMOs, such as termination engineering, heteroatom doping, defect engineering, and the formation of heterojunctions. The integration of MXenes with TMOs has addressed the current limitations of MXenes and significantly boosted OER activity. By considering these structural tuning parameters and limitation factors, researchers can gain insights into the design principles and optimization strategies for MXene- and TMO-integrated electrocatalysts. The review concludes with a summary of the key findings and an outlook on future research directions, emphasizing the unexplored potential and innovative approaches that could further advance the field of electrocatalytic water splitting.

## Abstract 2

This review summarizes recent advances in MXene and transition metal oxide (TMO) electrocatalysts for enhancing oxygen evolution reaction (OER), concluding with key findings and future research directions for further improvements.

## Graphical abstract

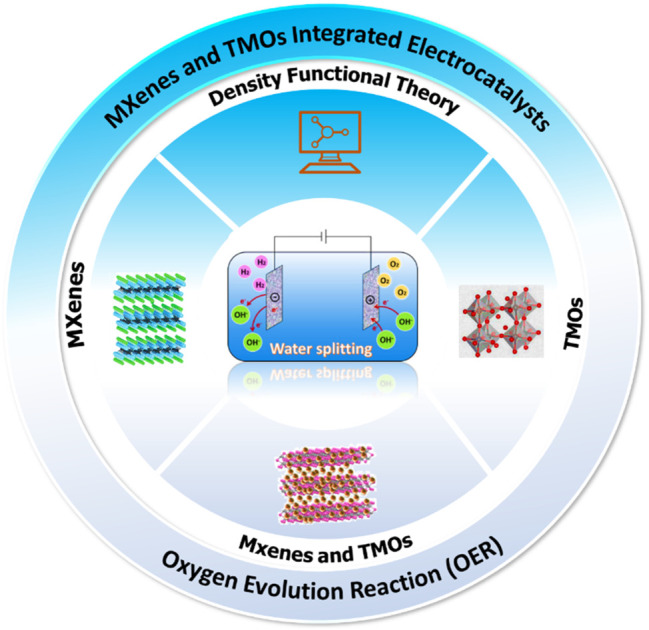

## 1. Introduction

Growing energy scarcity and environmental degradation have caused a lot of crises for the world community in recent decades.^1,2^ To fully utilise and harness the various renewable energy sources, including hydrogen, solar, wind, and tidal energy, tremendous efforts have been made. Hydrogen, a sustainable energy source, is viewed as a possible replacement for fossil fuels.^3,4^ Notably, water electrolysis to generate cathodic hydrogen and anodic oxygen is a timely and strategic approach to address the energy problem and concurrently reduce carbon emissions.^5^ In the electrochemical water splitting (EWS) process, two half-cell reactions occur, namely the hydrogen evolution reaction (HER) and the oxygen evolution reaction (OER).^6^ The OER is related to the four proton-coupled electron transfer processes and entails the production of oxygen–oxygen bonds.^7^ This results in sluggish kinetics, and consequently, demands a high overpotential. Therefore, it is imperative to use highly active electrocatalysts to facilitate the OER and lower the overpotential to minimise the energy loss that is inherent in these energy conversion devices.^8^ Noble metals, such as IrO_2_ and RuO_2_, can stimulate the proton-coupled charge transfer process and efficiently overcome slow kinetics up to this point. However, the main obstacles to their practical use are their high cost and scarcity.^9^ The need of the hour is a low-cost, readily available, earth-abundant, and noble metal-free catalyst that can accelerate large-scale industrial applications.

The most prominent materials in the field of electrocatalysis are transition metals (TMs), metal–organic frameworks (MOFs), and two-dimensional (2D) materials.^10–12^ Among other abundant materials, 2D materials are being extensively utilised as substitutes for precious metals.^13–17^ With a large specific surface area, strong hydrophilicity, tuneable structure, good metallic conductivity, high mechanical strength, and stability, MXenes feature a unique 2D layered structure resembling graphene.^18^ These qualities, together with their easy processing methods, have made them a popular option for energy conversion and storage applications. The standard formula for MXenes is M_*n*+1_X_*n*_T_*x*_ (*n* = 1, 2, or 3), where T denotes surface termination, X denotes carbon and/or nitrogen, and M denotes TMs. MXenes are usually prepared by extracting the “A” layers (A = Al, Si, Ga, *etc.*) from MAX precursors.^19^ MXenes attain a large surface area because of their layered structure, metallic and transitory electronic states that are brought about by TMs, and hydrophilic properties that are introduced by surface termination groups. Because of all these qualities, MXenes are effective electrocatalysts for the OER. Unfortunately, their catalytic activities are not as practical because most pristine MXenes restack readily and are unstable under oxidising conditions. Enhancing the intrinsic activity of the active sites or creating more catalytically active sites can enhance electrocatalyst performance.^20^ Motivated by these studies, several effective tactics have been put forth to increase the electrocatalytic activity of MXenes. These approaches include heterointerface engineering, hybrid engineering, surface termination engineering, and defect engineering.^21^ Furthermore, metal oxide catalysts are more stable in the OER in an alkaline medium.^22^ However, anion-exchange membrane electrolysis is still in its infancy as a technique, and the voltage efficiency and current density attained are not as high as those in acidic media electrolysis.^23^ Therefore, transition metal oxide (TMO)-based electrocatalysts, including spinel ferrites, perovskites, and layer-structured hydroxides, are considered due to their cost-effectiveness and high activity for practical utilisation. TMOs are among the most attractive candidates for water splitting because of several characteristics, including their remarkable electronic structure, and comparatively strong charge transfer properties even in the amorphous state. Even though most TMOs have a low specific surface area, low stability, limited metal sites, unmatchable electronic topologies, low availability of resources, and poor electronic conductivity, there is still a need for higher catalytic efficiency to meet industrial requirements. Consequently, enhancing TMOs' electrocatalytic activity needs to be given more attention. As a result, doping or heterostructuring has lately become a fascinating way to alter the catalysts' electronic configuration, resulting in an increase in active sites, an improvement in electrical conductivity, and the induction of a synergistic effect between TMO-based electrocatalysts during the entire water splitting process.^24^ Based on these superior properties, TMOs could be a desirable candidate for the preparation of MXene-based heterostructure composites and it is anticipated that the TMO functionality and adaptability may offer robust electrochemical performance for the OER.

The synthesis, characteristics, and possible electrochemical activity of MXenes have been the subject of several outstanding reviews. Nevertheless, there is no thorough and organised analysis of the latest developments in MXene and TMO-integrated electrocatalysts as electrode materials for the OER. This review provides a broad overview of pure TMOs, MXenes, and MXene-integrated TMO heterostructures for OER activity. To be more precise, we first describe the mechanism and factors affecting the OER activity. Different synthetic approaches of pure MXenes, TMOs, and MXene integrated TMO-based heterostructures are briefly discussed. Lastly, a quick overview of the potential of MXene integrated TMO electrocatalysts for future development is provided. In addition to offering motivation to progress in these fields, we anticipate that this study will provide insightful perspectives for developing cutting-edge MXene and TMO-integrated electrocatalysts for practical applications.

## 2. Oxygen evolution reaction in electrochemical water splitting

### 2.1 Overall water splitting

**Fig. 1:**
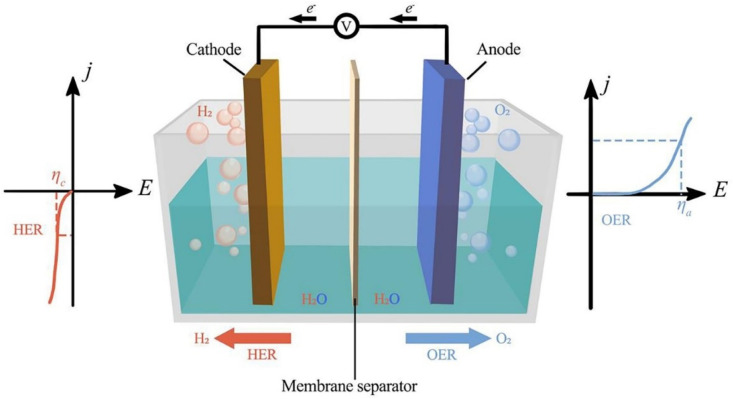
Electrochemical water splitting cell. Reproduced with permission from ref. 25. Copyright 2023, Elsevier.

Fig. 1 shows the three parts of traditional electrochemical cells for water splitting using bifunctional electrocatalysts: the anode, cathode, and aqueous electrolyte. Two kinds of reactions occur in electrochemical cells, namely water reduction and oxidation at the cathode and anode, respectively. Different electrochemical reactions occur at electrodes depending on the electrolyte, but the overall reaction is always the same.

Overall water splitting (OWS) reaction:1
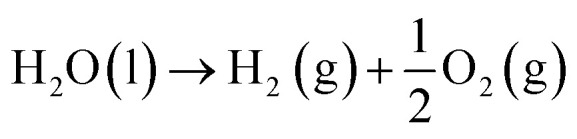


In an acidic electrolyte:

2 Cathodic: 4H^+^ + 4e^−^ → 2H_2_, *E*_c_ = 0.0 V

3 Anodic: 2H_2_O → O_2_ + 4H^+^ + 4e^−^, *E*_a_ = −1.23 V

In an alkaline electrolyte:

4 Cathodic: 4H_2_O + 4e^−^ → 2H_2_ + 4OH^−^, *E*_c_ = −0.83 V

5 Anodic: 4OH^−^ → O_2_ + 2H_2_O + 4e^−^, *E*_a_ = −0.40 V

In water splitting reactions, the theoretical thermodynamic value remains constant at 1.23 V at 25 °C and 1 atm, regardless of the reaction media used. A standard hydrogen electrode (SHE) is always used to calculate the *E*_c_ and *E*_a_ values. However, in contrast to the thermodynamic potential, a larger potential is often needed during the actual water splitting reaction.

### 2.2 Oxygen evolution reaction

**Fig. 2:**
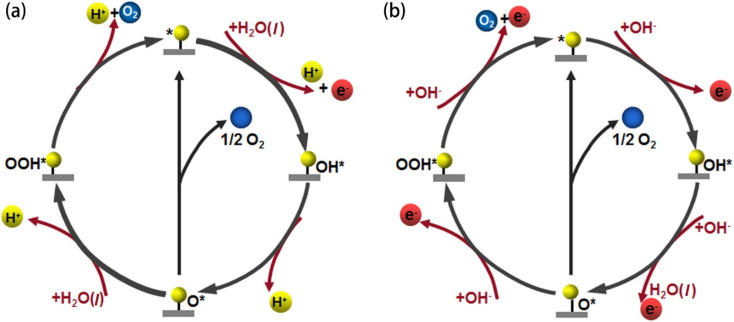
OER mechanism in (a) acidic and (b) alkaline media. Reproduced with permission from ref. 27. Copyright 2022, Elsevier.

The OER is a thermodynamically unfavourable process that needs a high potential to overcome the kinetic energy barrier since it involves the transfer of four electrons, involving the breaking of the O–H bond and the production of the O–O bond.^26^ The OER pathway differs in an alkaline and an acidic environment. Fig. 2(a and b) illustrate the mechanism of the OER in both acidic and alkaline media. Four proton-coupled electron transfer steps are involved in an acidic electrolyte to form an oxygen molecule after two water molecules are oxidised (eqn (11)). First, water is adsorbed on the active site (*), where it loses an electron and a proton to become adsorbed hydroxyl (OH*) (eqn (6)). After that, according to eqn (7), the OH* keeps losing protons and electrons, creating adsorbed oxygen (O*). From O*, there are two methods to produce O_2_. The first method produces O_2_ by directly combining two O* and releasing a free active site (eqn (8)). The second method involves O* and H_2_O interaction. A hydroperoxide intermediate (OOH*) is created by losing protons and electrons (eqn (9)). OOH* releases O_2_ and causes the active site (*) to regenerate after losing one more proton and one electron (eqn (10)).^27^

6 H_2_O(l) + * → OH* + H^+^ + e^−^

7 OH* → O* + H^+^ + e^−^

8 2O* → 2* + O_2_(g)

9 O* + H_2_O → OOH* + H^+^ + e^−^

10 OOH* → * + O_2_(g) + H^+^ + e^−^

11 Overall: 2H_2_O(l) → 2O_2_(g) + 4H^+^ + 4e^−^

where OH*, O*, and OOH* stand for the chemical species adsorbed on the active sites, (g) for the gas phase, and (l) for the liquid phase, and * for the reactive sites on the electrocatalyst's surface. In alkaline electrolytes, as opposed to acidic ones, oxygen molecules are created by converting OH^−^ through four stages of electron transfer. Furthermore, the production of the water molecule occurs (eqn (17)). To obtain OH*, an electron is first released by adsorbing OH^−^ on the active site (*) (eqn (12)). The produced OH* then reacts with OH^−^ to produce O* through the loss of an electron (eqn (13)). Alkaline electrolytes can produce O_2_ in two separate ways, similar to acidic electrolytes. One is that, according to eqn (14), two O* immediately combine to form O_2_ and release a free active site (*). An alternative method involves using OH^−^ to nucleophilically attack O* and produce an intermediate (OOH*) (eqn (15)). In addition to producing O_2_, the additional proton-coupled electron transfer of OOH* also realises the regeneration of the active site (*) (eqn (16)).^27^

12 OH^−^ + * → OH* + e^−^

13 OH* + OH^−^ → O* + H_2_O + e^−^

14 2O* → 2* + O_2_(g)

15 O* + OH^−^ → OOH* + e^−^

16 OOH* + OH^−^ → * + O_2_(g) + H_2_O(l) + e^−^

17 Overall: 4OH^−^ → 2H_2_O(l) + O_2_(g) + 4e^−^

In general, every basic step has a corresponding intermediate combination with a specified free energy, regardless of whether the conditions are alkaline or acidic. The reaction's theoretical point, *η* = G/e_1.23 V, is found at the step that has the largest free energy difference, known as the rate-determining step (RDS). Practically, the difference in free energy between HOO* and HO* has been found to be 3.3 ± 0.2 eV. The reference for assessing the catalytic activity of various catalysts was the difference in the binding free energy between O* and HO* (Δ*G*_O*_ − Δ*G*_HO*_). The slow reaction mechanism must be overcome by an ideal binding energy. IrO_2_ has been theoretically explained as a reasonably active metal oxide catalyst for the OER under acidic conditions based on reasonable binding energies to reaction intermediates.^28^ Based on its activity and stability under reaction circumstances, this catalyst has been experimentally demonstrated to be among the better OER catalysts available today; nonetheless, it is far from being an ideal OER catalyst in terms of activity, and it is not stable at high oxidative potentials.

### 2.3 Measurement parameters for assessing OER performance

This section critically discusses the important parameters to evaluate the suitability of materials for electrochemical activities concerning EWS reactions. The significant parameters are as follows:

#### 2.3.1 Onsite potential

The onsite potential is the potential where an electrocatalyst starts the desired reaction (like the OER).^29^ However, the significance of assessing the inherent capability of an electrocatalyst for the OER resides in its direct influence on the energy efficiency and economic viability of EWS.^12^ The onsite potential denotes the specific voltage at which the catalyst functions in practical settings, accounting for diverse environmental and operational variables having different electrolytes. Precisely quantifying potential aids in evaluating the actual efficacy of the catalyst, guaranteeing its ability to fulfil the requirements of industrial applications. Basically, linear sweep voltammetry (LSV) is an important technique for assessing the onsite potential.^30^ This method provides valuable information on the overpotential and current density, which are indicators of the catalyst's performance and effectiveness. Precise assessment of the potential on site is not only useful for comparing the performance of electrocatalysts but also guides the creation of novel materials with optimised features. This is essential for the advancement of sustainable technologies for H_2_ generation.

#### 2.3.2 Overpotential

Overpotential, represented by *η*, is a potential that is larger than equilibrium potential. The equilibrium potential of the HER is 0 V, whereas the equilibrium potential of the OER is 1.23 V. These numbers show that the HER needs less energy than the OER to overcome barriers. This additional potential is required for both the HER and the OER to initiate a reaction because of the intrinsic kinetic barrier. To assess electrode activity, overpotential has typically been computed at a specific current density. The overpotential is typically measured at a current density of 10 mA cm^−2^ in relation to the reversible hydrogen electrode (RHE). The electrode's electrochemical activity increases with decreasing *η* value.

#### 2.3.3 Stability

Stability, as an essential industrial application feature, is the capacity of a single catalyst to maintain activity over an extended period; chronoamperometry (CA) and chronopotentiometry (CP) are two main techniques that are usually available for investigating stability. These techniques evaluate stability in the presence of a specific potential or current density.^22^ CA tracks changes in current density. For instance, a density current of 10 mA cm^−2^ is typically used as a baseline when measuring catalyst stability; continuous performance for more than 10 hours shows favourable long-term stability. On the other hand, CP tracks the potential, respectively.^31^ In addition, the LSV technique is also used to compare the overpotential results difference before and after CV multiple cycles run. Multiple CV cycles are usually recorded to validate durability in terms of potential for the current whether the material(s) is stable or not.

#### 2.3.4 Turnover frequency

Turnover frequency (TOF) is a measure of the activity of a catalytic site under specific reaction conditions. It is defined as the number of reaction events (*i.e.*, molecules of hydrogen or oxygen evolved) per active site per unit of time. The formula for TOF is given by:18
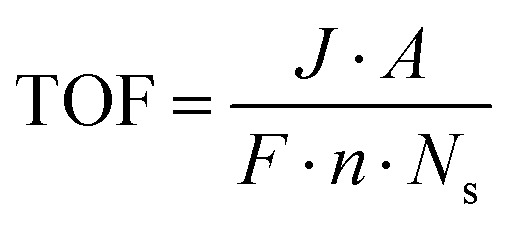
where *J* is the current density at a given overpotential (measured typically by LSV), *A* is the electrochemically active surface area of the electrode, *F* is Faraday's constant (96 485C mol^−1^), *N*_s_ is the number of active sites on the electrode, and *n* is the number of electrons transferred in the half-reaction (2 for the HER and 4 for the OER).

#### 2.3.5 Faradaic efficiency

The effectiveness of electron transfer supplied by the external circuit to support the electrochemical reaction is known as faradaic efficiency. It is also possible to think of it as the ratio of theoretical to experimental hydrogen production, which can be computed using the current density and a 100% faradaic yield. Therefore, the theoretical hydrogen production can be computed by integration from galvanostatic or potentiostatic electrolysis, while the experimental hydrogen production can be determined using gas chromatography (GC) or the traditional water–gas displacement method. The conventional water–gas displacement method is the most cost-effective. This entails creating a water-filled gas collection container and submerging the gadget above the electrode in the water. As electrocatalysis proceeds, the gas products gradually evolve and enter the container to replace the internal water. The GC methodology also quantifies product levels and is a more accurate method that allows real-time gas amount monitoring. The creation of byproducts or heat loss is the primary cause of faradaic loss, which is a useful metric for assessing the efficiency of electrocatalytic processes.

#### 2.3.6 Tafel slope and exchange current density

Higher current density coupled with a reduced overpotential is often indicative of a favourable catalyst. The Tafel slope can be calculated by using the formula *η* = *a* + *b* log *j*, where *b* is the Tafel slope and *j* is the current density. The reduced Tafel slope values indicate a notable increase in current density and a slight overpotential augmentation. Strong electrocatalytic activity and improved reaction kinetics are typically indicated by a lower Tafel slope. The catalyst's Tafel slope value decreases with increasing charge transfer capability. The exchange current density (*j*_0_) is typically another crucial component in determining the catalyst kinetics for the HER and OER. The ability to transport electrons under equilibrium conditions is represented by the value of *j*_0_, which is strongly associated with the intrinsic activity of catalysts. In general, the Tafel slope provides a good understanding of the principles of reactions, and the *j*_0_ indicates the catalysts' inherent activity. A low Tafel slope and a high *j*_0_ are necessary for an efficient and durable electrocatalyst.

#### 2.3.7 Double layer capacitance and electrochemically active surface area

Using a CV measurement, the double-layer capacitance (*C*_dl_) can be calculated. Plotting of CV curves as a function of different scan rates (*V*) is performed in a non-faradaic zone. The slope of the linear regression between current density differences in the middle of the prospective window of CV curves *versus* the scan rate can then be used to determine the *C*_dl_. It can be calculated using the following equation:19
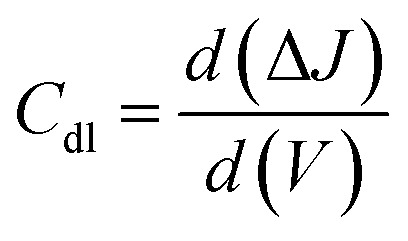


Based on the *C*_dl_ value, the electrochemically active surface area (ECSA) can be estimated using the following equation:20
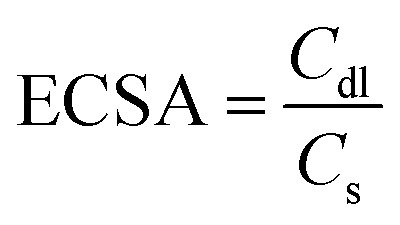
where *C*_s_ is the electrolyte's specific capacitance. Additionally, the ECSA is used to identify the aggregate binding sites for a certain electrode material on a given surface.^32^ A large ECSA often promotes the accumulation of water molecules and their derivatives, improving electrolyte contact and supplying a large number of reactive groups for catalytic performance interactions.^33^

#### 2.3.8 Electrochemical impedance spectroscopy

Typically, charge transfer resistance (*R*_ct_) is measured using electrochemical impedance spectroscopy (EIS), which offers a comprehensive view of the kinetics of electron transfer.^34^ For this purpose, the data are always plotted as Nyquist plots (*Z*_Re_*vs. Z*_Im_), Bode magnitude plots (frequency *vs.* |*Z*|) and Bode phase plots (frequency *vs.* phase). Subsequently, the data are fitted into an EIS circuit using Z-View software. Typically, an excitation signal V of modest amplitude alternating current (AC) voltage is superimposed over a steady direct current (DC) voltage to perform EIS. By applying a specific range of frequency (*f*) to an electrochemical cell and measuring the current flowing through it, one can derive an impedance spectrum.

#### 2.3.9 Mass and specific activities

The catalyst's activity for splitting water can also be determined by two other quantitative active parameters: mass and specific activities. The specific activity is the current normalised by the ECSA, which represents the inherent catalytic properties of the catalyst, while the mass activity, represented in amperes per gram (A g^−1^), is the current normalised by the catalyst loading. Similar to the TOF, the mass and specific activity must be obtained at a given overpotential. Specific activity is used to indicate the ECSA normalised current. Because the ECSA is more prone to catalyst loading, tailored action is more effective than mass activity. The ECSA will represent the underlying catalytic properties of the catalyst while normalising the current.

## 3. Synthesis methods of MXenes

**Fig. 3:**
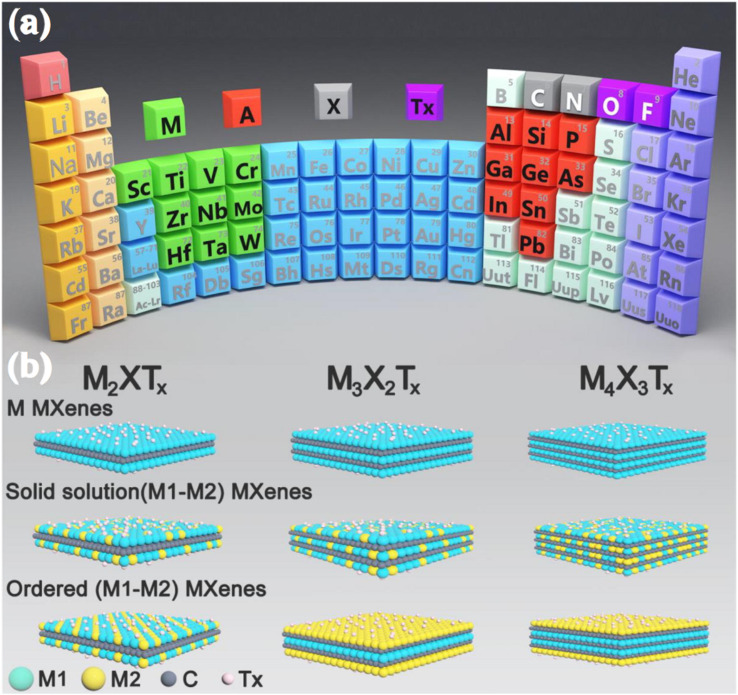
(a) The placement of the key components of the MAX phase on the periodic table of elements, and (b) arrangements and compositions of several MXenes, including M_2_XT_*x*_, M_3_X_2_T_*x*_, and M_4_X_3_T_*x*_. Reproduced with permission from ref. 38. Copyright 2021, Elsevier.

2D early transition metal carbides, nitrides, and carbonitrides, also known as MXenes, have gained significant attention in research since their discovery in 2011, making them the latest addition to the 2D nanomaterial family.^35,36^ MXene materials are characterised by the formula M_*n*+1_X_*n*_T_*x*_ (where *n* = 1, 2, 3).^37^ In this formula, “M” denotes a TM such as Zr, V, Nb, Hf, V, Sc, Nb, Ta, Ti, Cr, *etc.*, “X” may be either carbon or nitrogen, and “T_*x*_” refers to the terminal functional groups, namely –O, –F, and –OH (Fig. 3a).^39^ Until now, most MXenes have been produced by the targeted removal of the “A” layers (consisting of group IIIA or IVA elements) from MAX phases where M is an early transition metal, A is an A-group element (mostly groups 13 and 14), X is C and/or N. This process involves the use of hydrofluoric (HF) acid or a combination of lithium fluoride (LiF) and HF.^40^ Due to the detrimental impact of toxic HF on the environment, there has been growing interest in finding alternative preparation methods that are both environmentally friendly and efficient. Alkali treatment, Lewis acidic etching, electrochemical etching, water-free etching using polar organic solvents in conjunction with ammonium bifluoride, and other techniques have all been studied recently.^38^ In addition, several MXenes now in existence consist of multiple ‘M’ elements in solid solutions or ordered phases. These include (Ti, V)_2_CT_*x*_, (Ti, Nb)_2_CT_*x*_, (Cr, V)_3_C_2_T_*x*_, (Nb, Zr)_4_C_3_T_*x*_, (Mo_2_Ti)C_2_T_*x*_, and (Mo_2_Ti_2_)C_3_T_*x*_ (Fig. 3b).^38^ MXenes possess remarkable physicochemical properties due to their 2D ultrathin structure, distinctive electronic structures, and numerous surface termination groups. A high surface area, metallic conductivity, strong electrochemical stability, advantageous hydrophilic qualities, and modifiable surface chemistries are some of these attributes.^41^ Recently, there has been much research on MXenes, focusing on modifying their surface characteristics or combining them with other materials to fulfil the needs of various energy-related applications.^42^

**Fig. 4:**
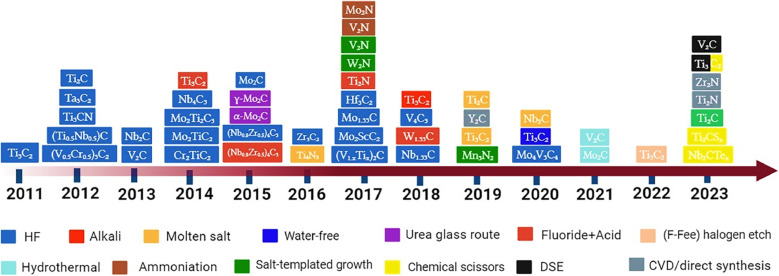
Overview of various kinds of MXenes, their methods of production, and the corresponding timeframes. Reproduced with permission from ref. 45. Copyright 2023, Elsevier.

Researchers have successfully acquired 25 or more MXenes by the targeted removal of a small number of atomic layers from nitride, carbide, and carbonitride pre-treatment agents using selective chemical etching.^43^ Etchants can be categorised into two groups: those that employ various ions and those that employ fluorine aqueous salts. During the early phases, MXenes are separated from MAX components by a process of fully immersing MAX systems in certain acids and breaking down M–A bonds. In 2011, the initial phase of synthesis of this 2D material took place, and various strategies were employed, including precursors, temperature conditions, and reaction time.^44^ Overall, the MXene synthesis route can be seen in Fig. 4. Furthermore, the types of synthesis routes are briefly discussed:^45^

### 3.1 HF etching

**Fig. 5:**
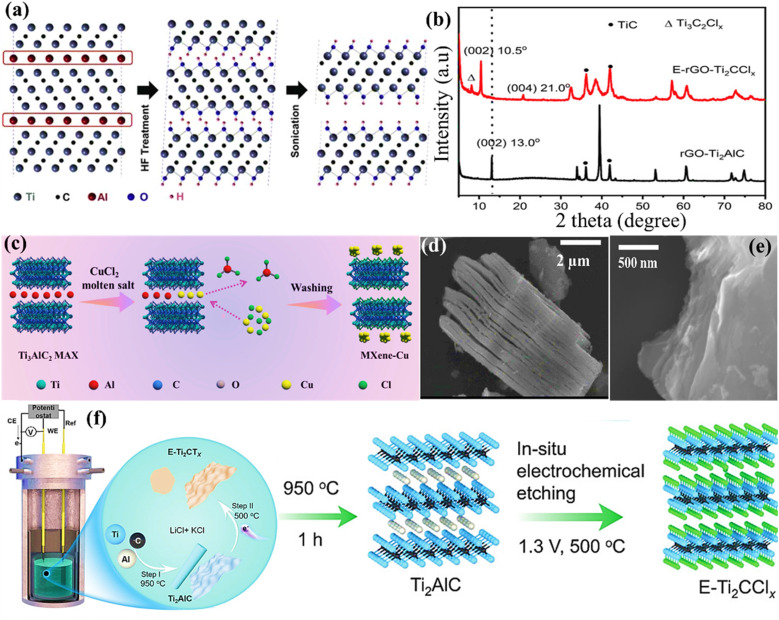
(a) Diagram illustrating the procedure for the synthesis of MXene Ti_3_C_2_T_*x*_ using HF (to etch Ti_3_AlC_2_). Reproduced with permission from ref. 48. Copyright 2024, Elsevier. (b) The XRD patterns of rGO integrated Ti_3_C_2_ MXene. Reproduced with permission from ref. 49. Copyright 2022, Wiley. (c) Illustration depicting the molten salt shielding MXene production technique. Reproduced with permission from ref. 50. Copyright 2023, Wiley. The SEM graph shows the (d) MS-Ti_3_C_2_T_*x*_ MXene and (e) e-MS-Ti_3_C_2_T_*x*_ (gathered by filtering). Reproduction with permission from ref. 51. Copyright 2022. ACS. (f) Schematic diagram showing the step-by-step method of creating Ti_2_AlC and electrochemically etching MXene (E-Ti_2_CT_*x*_). Reproduced with permission from ref. 49. Copyright 2022, Wiley.

The commonly used 2D MXene possesses several advantages over other 2D materials, such as a high specific surface area and good processability.^46^ However, its production differs from other 2D materials because of the “M” and “A” elements' strong metallic bonds. During initial research, highly corrosive substances such as an aqueous solution of HF were used to break down the chemical bonds between titanium (Ti) and aluminium (Al). This process preferentially eliminated the layers containing Al, resulting in the formation of multilayered flakes of Ti_3_C_2_ following further exfoliation.^47^ The effective synthesis of Ti_3_C_2_ expands the potential for etching several MAX phases, since MAX phases are a diverse group. Gogotsi's group successfully formed 2D MXene sheets by adding Ti_3_AlC_2_ powder to a saturated HF solution. Through the use of geometry optimisation and XRD analysis on the treated Ti_3_AlC_2_ powder, Naguib *et al.*^48^ determined that the Al layers may be dissolved by the HF acid solution and substituted with functional groups (mostly –F and –OH), resulting in a framework having a chemical formula of Ti_3_C_2_ (Fig. 5a). Furthermore, MXenes with different compositions have been prepared through HF-based etching. For instance, Ouisse *et al.*^52^ reported the chemical exfoliation of V_2_AlC by using precisely specified square pillars with lateral dimensions ranging from 7 μm to 500 μm. These pillars are fabricated from V_2_AlC single crystals several cm in size. HF penetration and the depletion of Al species occur mostly near borders. The following significant findings are reached by the authors through the fabrication of single-crystalline pillars of varying dimensions and the analysis of their transformation into MXenes as a function of time and position during etching in 49% HF: (i) HF penetration and etching essentially take place through facets oriented perpendicular to the basal planes, the latter being mostly immune to HF penetration. (ii) At distances greater than 40 μm, the etching rate slows down significantly from its initial linear rate of 2.2 ± 0.3 μm h^−1^. (iii) The HF process gradually causes partial carbonisation of the converted layers, which is a serious worry if one wants to maximise the convertible crystal size.^52^

### 3.2 *In situ* HF forming etching

Despite the widespread usage of the HF etching process in the manufacturing of Ti_3_C_2_T_*x*_, its practical use is significantly hindered due to the toxicity and destructive nature of HF.^53^ When comparing the use of a concentrated HF solution directly with the *in situ* production of HF, it is found that the latter can dissolve the A atom layers *via* a comparable reaction pathway. This indicates that protons and fluoride ions are essential for etching Ti_3_AlC_2_ powder.^54^ In addition, positively charged ions (such as Li^+^ and NH^4+^) present in the etching solution may insert themselves between layers, causing an expansion of the gap between the layers of MXene. This expansion weakens the contact between the MXene layers, making additional exfoliation easier and preventing the layers from restacking throughout the assembly process. Wang *et al.*^55^ used a straightforward hydrothermal method to develop multilayered Ti_3_C_2_T_*x*_ and examined how the combination of reactants, reaction duration, and reaction temperature influenced the amount of product obtained. Instead of using the direct HF solution etching procedure, they incorporated Ti_3_AlC_2_ particles into an NH_4_F aqueous solution and heated it at 150 °C for 12 h. During this procedure, NH_4_F would undergo slow hydrolysis, producing HF, which is used for etching the additional powder. Sheets of Ti_3_C_2_T_*x*_ with a size ranging from 4 to 15 μm and surfaces that are free from defects would be produced. Ghidiu *et al.*^56^ proposed a novel technique for preparing MXene colloidal solution, which has low toxicity and high yield. This approach utilises water as the primary solvent and a composite etchant consisting of HCl and a fluoride salt (typically LiF). The first step was the introduction of LiF particles into the HCl solution, which was then followed by the gradual addition of Ti_3_AlC_2_ powder. Lastly, a water bath was used to heat the mixture. X-ray diffraction (XRD) analysis was carried out to confirm the successful production of MXene (Fig. 5b). Through another study, the Ti_2_AlC MAX and Ti_2_CT_*x*_ MXene obtained from rGO are referred to as rGO-Ti_2_AlC and E-rGO-Ti_2_CClx, respectively. The MAX and MXene materials based on rGO exhibit distinct diffraction peaks (Fig. 5b),^49^ indicating a high level of crystallinity. Nevertheless, there is a little spike at 8° in E-Ti_2_CCl_*x*_, indicating the presence of the Ti_3_C_2_Cl_*x*_ phase. This phenomenon may be attributed to the partial conversion of Ti_2_AlC into Ti_3_AlC_2_, which has been previously seen to undergo a facile change throughout the annealing process.^49^ The addition of water and/or cations (Li^+^) to the hydrophilic and negatively charged MXene sheets also resulted in greater yields. Subsequent studies with this chemical etching agent have demonstrated that the lateral dimensions and surface flaws of the MXene sheets are highly influenced by the amount of LiF and the sonification treatment used (or their absence).^57^

### 3.3 Molten salt etching

Using molten salts in the process of etching improves the effectiveness and precision of separating MAX layers, leading to MXenes with exceptional qualities for a range of uses, including energy storage, sensors, and catalysis.^58^ Molten salt etching is characterised by its efficiency, safety, and simplicity, making it a promising technique for industrial applications.^59^ The molten salt etching method is a continuous process that does not require any intervention or chemical exposure, in contrast to HF-based procedures. Furthermore, the surface termination and interlayer spacing of MXenes can be engineered with the application of molten salt treatment. More crucially, mixed surface terminations and even bare surfaces that are challenging to realise in HF-based solutions can be obtained by using molten salt aided procedures. Li *et al.*^60^ have recently proposed a Lewis acidic molten salt etching technique as a substitute for conventional HF-based solvents in the production of MXenes. The authors have successfully created an accordion-like MXene structure that can be further exfoliated into high-quality nanosheets by adding the LiF additive into a molten-salt medium. Additionally, they discovered the method relies on the chemical interaction between –F groups and the tetrabutylammonium hydroxide (TBAOH) intercalation agent, through which LiF is incorporated into the molten salt media to promote effective exfoliation of the MXene. It is essential to mention that when the MXene is etched with a surficial “–F” layer, it might cause inadequate ion diffusion, which can negatively impact the efficiency of energy storage devices. Nevertheless, this restriction may be bypassed by using the molten salt technique. In addition, Yu *et al.*^50^ demonstrated the production of MXenes utilising the molten salt approach, using NaCl and KCl to exfoliate the Ti_3_AlC_2_ MAX phase (Fig. 5c). Regarding the morphology, scanning electron microscopy (SEM) investigation revealed a distinct difference between the original Ti_3_C_2_T_*x*_ structure, which had an accordion-like multilayered morphology (Fig. 5d), and the observed parallel restacked layer morphology (Fig. 5e). Li *et al.*^61^ have developed a cathode made of a composite of V_2_C (MXene) and nickel di-selenide (V_2_C@NiSe_2_). This cathode is created by removing the V_2_AlC MAX phase using Lewis acidic molten salts, followed by heating it with selenium (Se). The cathode material V_2_C@NiSe_2_ demonstrated reversible redox reactions, including converting Ni^2+^ to Ni^*x*+^ and Se^−^ to Se^*x*+^ throughout the charge–discharge process. Since Se can substitute functional groups on V_2_C, it may effectively prevent significant interactions across AlCl_4_^−^ and V_2_C layers, increasing their energy density. Regarding deeper morphology, after undergoing the selenization procedure, NiSe_2_ is effectively produced inside the V_2_C layers, decreasing surface groups like –Cl, –O, and –OH of the V_2_C layers.^61^ In another study, Li *et al.*^62^ conducted an investigation where they developed and confirmed the effectiveness of a redox-controlled A-site etching method for MAX phases in Lewis acidic melts. This method synthesised several MXenes using typical MAX-phase precursors, including A elements such as Si, Zn, and Ga. The molten salt synthesis approach yields a Ti_3_C_2_ MXene material with a negative electrode that can store up to 738C g^−1^ (205 mA h g^−1^) of Li^+^ with an excellent charge–discharge rate and an electrochemical signature similar to that of pseudocapacitors in a 1 M LiPF_6_ carbonate-based electrolyte.

### 3.4 Electrochemical etching

Electrochemical etching is an advanced method used to produce and modify MXenes. This technique involves using an electric current to specifically eliminate layers within MAX phases, creating high-quality, very thin MXene sheets.^63^ In addition, electrochemical etching has other benefits such as precise regulation of the etching process, the capability to generate films over a wide region, and the possibility to adjust the surface chemistry of MXenes. Previously, Simon *et al.*^49^ described an electrochemical etching technique (Fig. 5f) for the direct synthesis of Ti_2_C MXene using elemental substances (Ti, Al, and C). In this work, a variety of carbon sources are used, such as reduced graphene oxide (rGO) and carbon nanotubes (CNTs), in conjunction with Ti and Al micropowders. The goal is to produce Ti_2_AlC MAX with controlled 1D and 2D morphologies. This is achieved by *in situ* electrochemical etching, which converts Ti_2_AlC MAX into Ti_2_CT_*x*_ MXene. Etching is performed in an inexpensive lithium chloride–potassium chloride (LiCl–KCl) solution. Together with Cl termination, the addition of the O surface group through further treatment with ammonium persulfate (APS) may start the pseudocapacitive redox reaction that produces Ti_2_CCl_*y*_O_*z*_ in a non-aqueous electrolyte. In another study, Wong *et al.*^64^ devised a simple and efficient electrochemical etching technique to produce F-free and Cl-containing Ti_3_C_2_T_*x*_ compounds. This approach involves using a mixture of lithium hydroxide (LiOH) and LiCl in an aqueous solution, resulting in an etching efficiency of 92.2%. During the synthesis process, the use of sonification alone is sufficient to separate the layers of Ti_3_C_2_T_*x*_ without requiring any potentially harmful organic intercalant. The delaminated Ti_3_C_2_T_*x*_ flakes produced have a lateral dimension of around 3.8 μm and a thickness of approximately 3.9 nm. For at least 15 days, these flakes might be stable in an aqueous dispersion. The filtered Ti_3_C_2_T_*x*_ film has an electrical conductivity of 1663 S cm^−1^, a Young's modulus of 13.4 GPa, and a tensile strength of 20.5 MPa. It also shows that for supercapacitors, the respective capacitances are 323.7 F g^−1^, 1.39 F cm^−2^, and 1160 F cm^−3^.

### 3.5 Alkali etching

**Fig. 6:**
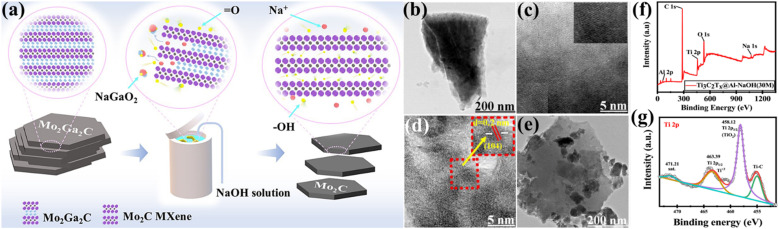
(a) Schematic illustration of alkali etching for Mo_2_C MXene production. Reproduced with permission from ref. 66. Copyright 2023, SciOpen. The TEM and HR-TEM images of the Ti_3_C_2_T_*x*_@Al–NaOH MXenes: (b and c) 25 M, (d) 30 M, and (e) 35 M. Reproduced with permission from ref. 67. Copyright 2024, Elsevier. (f and g) The XPS survey spectra of Ti_3_C_2_T_*x*_@Al–NaOH, as well as the high-resolution spectra of Ti 2p. Reproduced with permission from ref. 67. Copyright 2024, Elsevier.

The alkali etching procedure uses strong alkali solutions to selectively remove the ‘A’ layers from MAX phases, creating MXene structures that possess distinct characteristics.^65^ Alkali etching is well regarded for its effectiveness, simplicity, and capability to create MXenes with customised surface chemistries as well as geometries. Zhou *et al.*^66^ have conducted research in which they proposed a new technique for synthesising Mo_2_C MXene from Mo_2_Ga_2_C *via* hydrothermal etching with alkali solutions. The synthesis of very pure Mo_2_C MXene was achieved by effective etching using sodium hydroxide (NaOH), whereas attempts to etch using LiOH and potassium hydroxide (KOH) were unsuccessful. The concentration of NaOH, temperature, and duration significantly influence the purity of the as-prepared MXene. The synthesis of pure Mo_2_C MXene can be achieved by etching with 20 M NaOH at a temperature of 180 °C for a duration of 24 h. Following intercalation with hexadecyl trimethyl ammonium bromide at a temperature of 90 °C for 96 h, only a few layers of Mo_2_C MXene were produced (Fig. 6a).^66^ Que *et al.*^67^ provide a hydrothermal alkali etching method for synthesising Ti_3_C_2_T_*x*_@Al–NaOH (T_*x*_ = –OH, –O) MXene, avoiding the use of fluorine. This process involves the reaction of NaOH with a MAX solution. The Ti_3_C_2_T_*x*_@Al–NaOH sample, which was immersed in a NaOH solution for 15 h, has excellent electrochemical characteristics. It is possible to precisely alter MXene's surface functional groups, –O and –OH, by selecting the appropriate alkali-assisted etching technique. Fig. 6(b and c) show the transmission electron microscopy (TEM) and high resolution-TEM (HR-TEM) images of Ti_3_C_2_T_*x*_@Al–NaOH (25 M) MXene. Fig. 6(d and e) show the images of Ti_3_C_2_T_*x*_@Al–NaOH (30 M) MXene, which reveal lattice fringes of 0.2 nm for the (104) plane.^67^Fig. 6(f and g) display the X-ray photoelectron spectroscopy (XPS) spectra of Ti_3_C_2_T_*x*_@Al–NaOH (30 M) MXene. The spectra demonstrate the presence of Al 2p peaks in the energy range of 65–85 eV, C 1s peaks in the range of 280–290 eV, Ti 2p peaks in the range of 450–470 eV, O 1s peaks in the range of 525–540 eV, and Na 1s peaks in the range of 1060–1078 eV. The absence of an Al signal indicates that the Al has been successfully removed from the precursor material. The deconvolution of the Ti 2p spectra within the energy range of 450 eV to 470 eV indicates the presence of Ti–O chemical bonds. This implies that partial surface oxidation may have led to the development of –O surface groups.^67^

### 3.6 Ionic liquid and microwave-assisted etching

**Fig. 7:**
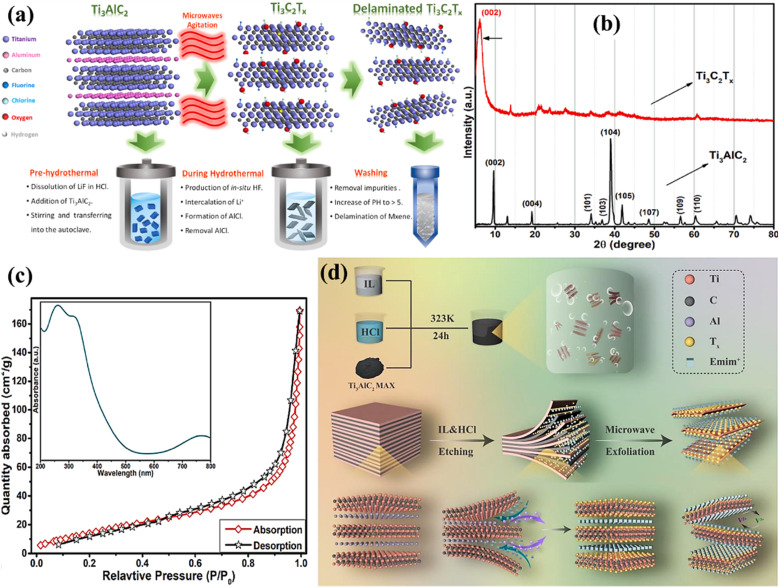
(a) The process of obtaining Ti_3_C_2_T_*x*_ MXene from Ti_3_AlC_2_*via* the microwave-assisted hydrothermal technique. Reproduced with permission from ref. 70. Copyright 2022, Elsevier. (b) The XRD spectra illustrating the conversion of the Ti_3_AlC_2_ MAX phase to Ti_3_C_2_T_*x*_ (MXene). Reproduced with permission from ref. 70. Copyright 2022, Elsevier. (c) The BET curve and UV findings (inset) of Ti_3_C_2_T_*x*_. Reproduced with permission from ref. 70. Copyright 2022, Elsevier. (d) Schematic diagram of ionic liquid etching of Ti_3_C_2_ MXene. Reproduced with permission from ref. 71. Copyright 2023, Elsevier.

Ionic liquids are used as the etching medium in this novel etching of the MAX phase approach. These liquids provide a stable and easily adjustable environment that improves the selectivity as well as the effectiveness of the etching procedure.^68^ Utilising ionic liquids has several advantages, such as low vapour pressure, exceptional thermal stability, and the capacity to dissolve various substances. The characteristics of these materials enable the accurate elimination of the ‘A’ layers from MAX phases while preserving the structural integrity of the final MXene.^69^ In addition, microwave irradiation speeds up the etching process by quickly and evenly heating the substrate, resulting in a considerable reduction in processing time compared with conventional techniques. Through microwave-assisted synthesis, Numan *et al.*^70^ used a new microwave-assisted hydrothermal technique to remove Al from the MAX phase and separate it during a processing period of 2 h (Fig. 7a). This approach significantly reduces the time required from a maximum of 48 h to only 30 minutes and lowers the temperature from 180 to 40 °C. The findings demonstrated the production of a superior MXene material with little Al residue, hence confirming the effectiveness of the existing methodology. This study not only develops a thorough procedure for quickly producing large amounts of high-quality MXene, but also creates opportunities for its widespread use in commercial applications. Two separate peaks can be seen in the Ti_3_AlC_2_ XRD spectra at 2*θ* values of 9.6°, 19.2°, 34.1°, 38.8°, 41.9°, 48.5°, 56.5°, and 60.4°. These correspond to the lattice planes of Ti_3_AlC_2_ (JCPDS No. 52-0875), such as (002), (004), (101), (103), (104), (105), (109), and (110). After the hydrothermal treatment with the use of microwaves, the distinct peaks from the Ti_3_AlC_2_ pattern at 20°, 34.1°, 36.8°, and 41.9° were nearly completely erased, and a broad strong peak appeared at 6.2°, which could be related to the (002) reflection of Ti_3_C_2_. The (002) peak of the precursor Ti_3_AlC_2_ is seen at an angle of 9.7°. However, after undergoing etching by microwave-assisted hydrothermal treatment, the peak has shifted to a lower angle of 6.2° (as shown in Fig. 7b, Ti_3_C_2_T_*x*_ spectrum). Fig. 7c displays the absorption–desorption Brunauer–Emmett–Teller (BET) curves and the UV-visible absorbance spectrum (inset) recorded for Ti_3_C_2_T_*x*_ MXene. The BET curve of Ti_3_C_2_T_*x*_ exhibited a clear hysteresis loop due to the presence of slit-shaped pores between the neighbouring layers. This indicates that Ti_3_C_2_T_*x*_ shows type IV nitrogen adsorption isotherms. The UV-visible spectrum exhibits three distinct peaks at around 261, 323, and 770 nm, which are due to the presence of the Ti_3_C_2_T_*x*_ MXene derived from the MAX phase and mostly documented in the literature. Fang *et al.*^71^ developed new ionic liquid microwave (IL-MW-MXene) Ti_3_C_2_T_*x*_ with a distinct 2D structure and a high concentration of active functional groups. This material was prepared using a combination of ionic liquid etching along with microwave-assisted stripping (Fig. 7d). It is suggested that this material has great potential as an electrocatalyst for V^3+^/V^2+^ redox reactions.

## 4. Synthesis methods of integrated MXenes and TMOs

There are various methods for the synthesis of MXene integrated TMOs for their usage in a wide range of applications.^72^ Due to its advantages, such as high product crystallinity, low operating temperatures, and high diffusion rates, the hydrothermal method has drawn a lot of attention. Additionally, MXene-based heterostructures' surface functional groups can be manipulated to increase their activity.^73^ Huang *et al.*^74^ revealed three nanocomposites based on V_2_O_5_/MXene with various V_2_O_5_ NP morphologies prepared using a straightforward hydrothermal technique. The specific capacitances of nanoribbon V_2_O_5_ on MXene, nanocuboid V_2_O_5_ on MXene, and nanosphere V_2_O_5_ on MXene were 114 F g^−1^, 180 F g^−1^, and 217 F g^−1^, respectively, in 1.0 M LiNO_3_ aqueous electrolyte, which were significantly higher than the 15 F g^−1^ of MXene. Han *et al.*^75^ showed the detailed interaction between hexagonal and monoclinic WO_3_ and Ti_3_C_2_. Mono-WO_3_–Ti_3_C_2_ and hexa-WO_3_–Ti_3_C_2_ hybrids were produced in a single step hydrothermal method by carefully regulating the WO_3_ phase. When compared to monoclinic and hexagonal WO_3_, both WO_3_ hybrids possessed a surface area that was three to four times larger, allowing for easier ionic mobility. In terms of size and shape, self-assembly has emerged as the most sophisticated bottom-up method for creating nanomaterials. This technique results in closely packed metal-oxide nanostructures on MXene nanosheets and is inexpensive, simple to deploy, and very effective.^73^ van der Waals interactions, or electrostatic interactions, cause TMOs to self-assemble atop MXenes (Ti_3_C_2_), lowering the surface energy and stabilising the structure in the process. Wang *et al.*^76^ revealed a flexible technique for creating flower-shaped Co-based bimetallic oxide heterostructures that can be utilised to electrostatically self-assemble MXene nanosheets to create a three-dimensional crossing network topology. The constructed network can be used to create conductive channels that allow electrons to flow freely. With the least amount of reflection loss, the resulting bimetallic MXene composite showed outstanding microwave absorption capability. This self-assembly method does not cause the structural degeneration of MXene nanosheets, in contrast to hydrothermal production. Saraf *et al.*^77^ produced free-standing films with varying compositions based on an α-MoO_3_/Ti_3_C_2_ heterostructure through self-assembly at room temperature without the need for binders. The mechanical stability and effortless synthesis of free-standing films with α-MoO_3_ are offered by Ti_3_C_2_ MXenes. Chemical liquid phase deposition allows targeted coatings to be applied to a material through carefully regulated chemical reactions. This method can be applied to MXene nanosheets to prevent restacking or to peel away the inner layers of multilayered MXenes.^73^ Li *et al.*^78^ uniformly bonded RuO_2_ nanoparticles to MXene nanosheets. Before the Ru cations oxidised during the reaction, negatively charged MXene surfaces electrostatically self-attracted onto positively charged Ru^3+^ surfaces, thereby preventing the restacking of MXene nanosheets. The enhanced specific surface area of RuO_2_·*x*H_2_O@MXene mesoporous structures may have accelerated electrolyte ion migration and diffusion, improving electrochemical performance even further. Dong *et al.*^79^ have shown how to create MnO_2_/Ti_3_C_2_T_*x*_ nanocomposites using a light chemical deposition technique. 1D MnO_2_ nanoneedles and 2D MXene sheets were combined to create a unique structural electrode material for flexible supercapacitors. Yang *et al.*^80^ prepared a novel MnO_2_/p-Ti_3_C_2_T_*x*_ nanocomposite-based film through a vacuum filtration process, which was employed directly as a pseudocapacitive electrode. Wang *et al.*^81^ demonstrated the integration of delaminated MXene sheets with pseudocapacitive MoO_3_ nanobelts *via* a vacuum-assisted technique. The MoO_3_/MXene hybrid film's demonstrates how MoO_3_ nanobelts are positioned in between the conductive MXene layers to stop MXene from restacking. The resulting construction increased the energy storage capacity's active surface.

## 5. Strategies for tuning the structures of MXenes and TMOs

**Fig. 8:**
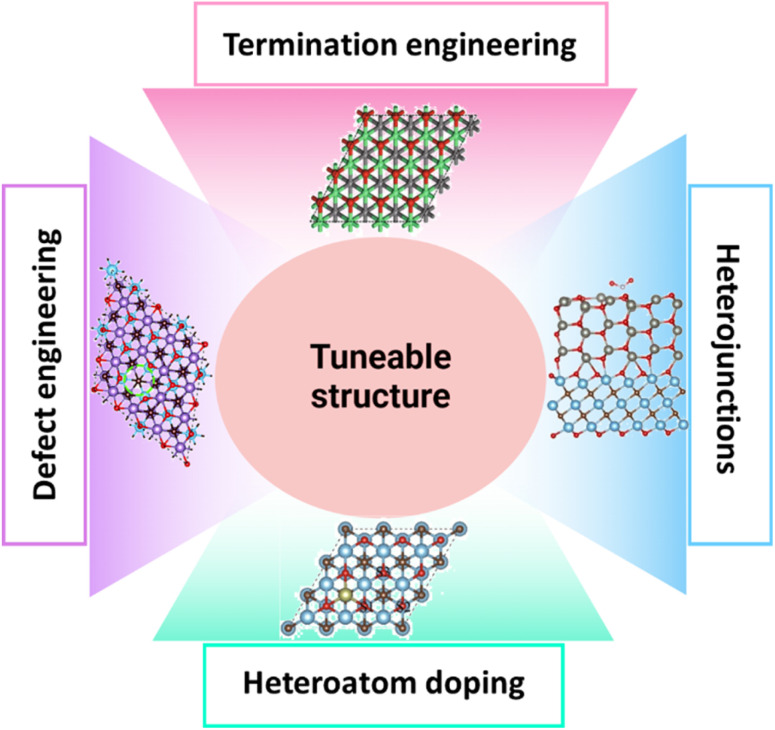
Graphic illustration for different strategies for tuning MXene-based TMOs to enhance electrocatalytic performance, including termination engineering, defect engineering, heteroatom doping, and heterojunction formation.

The tuneable structure of various nanomaterials (MXenes and TMOs) is essential for improving the effectiveness and functionality of electrocatalysts for OWS, including both the HER and the OER.^22,82,83^ The majority of electrochemical conversion events necessitate intimate interactions between reactants and catalyst surfaces. Catalyst surface engineering techniques are often essential for obtaining the intended activity. Therefore, it is generally accepted that the main method for altering the characteristics and catalytic activity of MXenes is surface engineering. The surface of MXenes is engineered using a variety of techniques. First, it has been shown that MXenes often have particular T_*x*_ terminations, which have a significant impact on their hydrophilic properties, electronic structures, stability, and catalytic activity. Thus, the simplest approach to engineering MXenes is often to modify the surface T_*x*_ terminations. An additional tactic involves manipulating surface imperfections in MXenes that arise from the etching or exfoliation procedures, which significantly influences their electrical and surface characteristics. As a result, heteroatom doping which involves the insertion of metal and non-metal is likewise thought to be beneficial for changing the characteristics of MXenes. Finally, it has been demonstrated that adding secondary materials to MXenes is a promising approach to change all the composites' characteristics. Researchers can enhance catalytic activity, stability, and overall efficiency by accurately manipulating the size, shape, composition, and surface characteristics of nanomaterials (electrocatalysts). A brief discussion on different aspects of tuneable structures is given ahead (Fig. 8).

### 5.1 Termination engineering

MXenes' surface chemistry is intricately linked to the surface termination groups (also known as termination engineering).^84^ Recently, Talapin's group synthesised MXenes with a range of surface terminal groups, including O, NH, S, Cl, Se, Br, and Te.^85^ This engineering area is dedicated to manipulating the outermost layers of atoms, referred to as surface terminations, to improve the catalytic capabilities for specific reactions, such as the OER in electrochemical water splitting reactions. Chen *et al.*^86^ provided a straightforward and innovative approach to modify the surface structure of few-layered MXene flakes by incorporating a tiny quantity of Nb element. Due to the strong attraction between the elements Nb and O, V_1.8_Nb_0.2_CT_*x*_ and Ti_2.7_Nb_0.3_C_2_T_*x*_ MXenes have a much smaller number of –F functional groups and a greater amount of O compared to V_2_CT_*x*_ and Ti_3_C_2_T_*x*_ MXenes, respectively. Both V_1.8_Nb_0.2_CT_*x*_ and Ti_2.7_Nb_0.3_C_2_T_*x*_ MXenes exhibit improved pseudocapacitance performance. Hence, the findings in this study are relevant to manipulating surface properties and systematically creating 2D MXene materials to transform them into highly promising and economically viable electrode materials for supercapacitors or other energy storage devices. Furthermore, Cheng *et al.*^87^ have shown that (111)-facet Co_3_O_4_ nanosheets have a unique surface termination consisting only of edge-sharing octahedral Co^3+^ ions, similar to CoOOH. These nanosheets exhibit a much greater current density at 1.63 V (*versus* RHE) compared to commercial RuO_2_, with an estimated 40 times increase. It has been discovered that this surface termination exhibits an oxidised oxygen state, unlike typical Co–O systems. This oxidised state may function as an independent active site, therefore surpassing the limit imposed by the scaling relationship. This study advances the use of oxide electrocatalysts in the energy conversion field using the surface termination engineering technique.

### 5.2 Heteroatom doping

Heteroatom doping greatly increases the effectiveness of catalysts for the OER by altering their electronic structure, generating additional active sites, and enhancing structural stability.^88^ Introducing heteroatoms, such as TMs or non-metals, into the catalyst's lattice makes it possible to enhance the binding energies of OER intermediates. This is achieved by modifying the charge distribution and band structure, improving charge transfer and lowering overpotentials. Geng *et al.*^89^ created a liquid suspension consisting of single-layered nitrogen-doped Ti_3_C_2_ sheets that exhibit exceptional uniformity and durability. The maintenance of the hydrophilic surface of the host layers during the annealing process is critical for accomplishing this goal. The sample's capacitive electrochemical performance was significantly improved due to enhanced electrical conductivity and redox reactivity brought about by nitrogen doping of individual MXene monolayers. Zhang *et al.*^81^ provided a simple solvothermal method for creating a binder-free Fe–S–NiMoO_4_/MoO_3_ electrode using an array of evenly produced nanorods on nickel foam (NF). The findings show that Fe doping can change the electrical structure, control the MoO_3_ crystal plane, and increase conductivity. This work offers a useful method for designing electrocatalysts for future industrial uses. Moreover, dopants have the ability to establish defect sites and create synergistic interactions that improve catalytic activity. The catalyst's longevity and efficiency are improved under operating settings due to structural enhancements, including induced lattice strain and greater corrosion resistance. This deliberate alteration at the atomic scale allows for the creation of more efficient and resilient OER catalysts, which are essential for the progress of sustainable energy technology.

### 5.3 Defect engineering

Defect engineering enhances the efficacy of catalysts for the OER by adding vacancies, dislocations, and other deficiencies to the catalyst's structure, significantly improving its catalytic efficiency.^90^ These imperfections generate extra active sites and modify the electronic characteristics of the material, resulting in enhanced adsorption and activation of OER intermediates. Researchers can improve the performance of catalysts in OER applications by precisely manipulating their defects. In this regard, Yamauchi *et al.*^91^ found that although the intermediates coordinated with –H form whole molecules like *HCOOH and *H_2_CO, the intermediates coordinated with –C take the form of fragments like *COOH and *CHO. Remarkably, transition-metal vacancies strengthen the binding of fragment-type intermediates to the majority of MXenes. Sun *et al.*^92^ created a multi-functional MXene fibre by using defect engineering techniques and wet spinning to combine MoO_3−*x*_ nanobelts with lots of oxygen vacancies. The generated MXene/MoO_3−*x*_ fibres, which benefit from the abundance of oxygen vacancies, increase the number of active sites for interaction with electrolyte ions and significantly increase the rate capacities of the fibres because of the larger spacing between MXene flakes. Moreover, Lu *et al.*^93^ conducted a study where they used an electrochemical lithiation approach to create oxygen defects in NiCo_2_O_4_ nanorods. By adjusting the *in situ* lithiation potentials, it is possible to effectively control the concentration of oxygen defects and the resulting catalytic activity. Furthermore, the correlation between defect density variations and electrical structures and the lithiation cut-off voltages is unveiled. The results show that a two-step conversion process and intercalation occurred in NiCo_2_O_4_ nanorods. In particular, the lithiation-induced reaction produced a CoO@NiO-based structure with a lower oxidation state and a higher defect density. As a result, the faulty CoO@NiO-based catalyst exhibits exceptional OER performance, needing just a 270 mV overpotential to reach a 10 mA cm^−2^ current density. This is about 74 mV lower than the initial nanomaterials. This study presents an innovative approach to investigate catalysts with excellent performance and structural stability while controlling defects.

### 5.4 Heterojunctions

Heterojunctions are critical for improving the effectiveness of electrocatalysts towards the OER.^94^ By integrating many different substances at the nanoscale, heterojunctions provide interfaces that greatly enhance the efficiency of charge separation and transmission.^95^ These interfaces often result in the creation of inherent electric fields and band alignment, which aid in the transportation of electrons and holes towards active sites, which can decrease the overpotential needed for the OER. Furthermore, the interaction between diverse materials in heterojunctions may amplify the inherent catalytic activity and stability of the electrocatalysts. For instance, Chu *et al.*^96^ used an *in situ* hydrothermal method to effectively produce distinct endogenous hetero-MXenes consisting of amorphous MoS_2_ coupled with fluoride-free Mo_2_CT_*x*_ (hetero-Mo_2_C) directly from Mo_2_Ga_2_C MAX. Adding MoS_2_ to hetero-MXenes results in a unique shape and heterojunction, giving them exceptional stability and enhancing their capacity to store Li^+^ ions. This also improves their ability to transfer charges and adsorb lithium ions. Furthermore, Wang *et al.*^97^ reported Co_3_O_4_ nanowires coated with NiFe-LDH (layered double hydroxide) nanoparticles, resulting in the formation of many heterogeneous interfaces. This results in a notable enhancement of the surface area and permits the modification of the electron density distribution. The resulting composite includes a hybrid crystalline/amorphous NiFe-LDH phase and many unsaturated Co sites on Co_3_O_4_. Both parts offer many active sites and improve the flow of electric charge. Co–Ni–Fe electron transport channels are generated at the core–shell heterojunction, allowing electrons to go from Co_3_O_4_ to NiFe-LDH. The Co_3_O_4_ @NiFe-LDH/NF-100 catalyst exhibited efficient OER performance, with a low overpotential of 270 mV at a current density of 50 mA cm^−2^. Additionally, it demonstrated exceptional stability in alkaline environments. This conventional core–shell heterojunction facilitates the dissociation of H_2_O and enhances the adsorption of intermediates, enhancing the OER process.

## 6. MXenes as electrocatalysts for the OER

### 6.1 MXenes for the OER

MXenes, a recently developed group of 2D transition metal carbides, nitrides, and carbonitrides, have attracted considerable interest as electrocatalysts for the OER because of their distinctive structural and electrical characteristics. MXenes are formed by selectively removing the A-layer from MAX phases. In this context, M refers to an early TM, A refers to an element from group 13 or 14, and X indicates either carbon (C) or nitrogen (N). The etching procedure yields a layered structure with a large surface area, adequate active sites, and adjustable surface chemistry, all necessary for electrocatalytic applications. MXenes possess a high electrical conductivity due to their metallic-like properties, which allows for efficient electron transfer during the process of the OER. In addition, the hydrophilic surfaces of MXenes, which are often terminated with functional groups like –OH, –O, or –F, enable easy dispersion in water-based solutions and promote favourable contact with water molecules, and this interaction is an important factor for the OER. The existence of these functional groups might also alter the electrical characteristics of MXenes, enhancing the binding energies of reaction intermediates. The composition and structure of MXenes can be modified, resulting in adjustable catalytic capabilities. The electronic structure of MXenes may be altered to improve catalytic activity by adjusting the TM and modifying the surface terminations. Importantly, the spacing between layers in MXenes may be modified by introducing various ions or molecules, enhancing their catalytic capabilities *via* improved access to active sites. For example, Ti_3_C_2_T_*x*_ MXenes have shown potential as OER electrocatalysts because they may provide ideal binding sites for oxygen intermediates. To date, various categories of MXenes such as Ti_2_C,^98^ Ti_3_C_2_T_*x*_,^99^ Mo_2_C,^100^ Mo_2_TiC_2_,^101^ Mo_2_Ti_2_C_3_,^102^ Nb_2_C,^103^ Cr_2_C,^104^ V_2_C,^105^ and so on have been reported for water splitting applications. Furthermore, MXenes can be combined with other materials to create heterostructures, which enable the combination of the beneficial characteristics of each component. For instance, combining MXenes with metal oxides, phosphides, or sulphides can create hybrid structures that exhibit synergistic effects, resulting in improved activity and stability for the OER. These composite materials can leverage the excellent conductivity and numerous active sites provided by MXenes, as well as the inherent catalytic characteristics of the other components.

Despite the widespread use of MXenes in different practical sectors, the use of MXene-based products is still restricted owing to their inherent instability. The primary exhibition of MXenes' instability includes flake oxidation, colloidal solution instability, swelling, and thin film deterioration. Efforts to investigate stability approaches have increased since MXene materials are needed for their stability. Recently, a significant amount of research has been conducted on methods to stabilise MXenes. Their motive is to increase the shelf life of MXenes and improve their characteristics. Additionally, these studies aim to make it easier to use MXenes in many fields such as catalysis, energy storage, and biomedicine. Several ways have been employed to efficiently stabilise MXenes, such as optimising the synthesis of the MAX phase, modifying the MXene preparation procedure, regulating storage conditions, and deploying shielding measures: (i) improving the stability and electrical conductivity of MXenes through optimisation of the MAX phase synthesis is a workable strategy that has a significant impact on the synthesis process and the functionality of the final products. Mathis *et al.*^106^ discovered that introducing an excessive amount of Al during the Ti_3_AlC_2_ MAX phase precursor production led to Ti_3_AlC_2_ grains with enhanced stoichiometry and crystallinity. The presence of molten metal in sintering led to an improved diffusion of reactants. Consequently, the durability and characteristics of the altered Ti_3_C_2_, which was etched from improved Ti_3_AlC_2_, exhibited notable stability. (ii) The shape and size of MXenes have profoundly impacted both their characteristics and uses. Specifically, the dimensions of the sheet of few-layer 2D materials significantly influence their rate of deterioration when immersed in a liquid solution. In their study, Zhang *et al.*^107^ produced Ti_3_C_2_T_*x*_ MXene films by isolating Ti_3_C_2_T_*x*_ sheets from a colloidal dispersion using vacuum filtering. The resulting MXene films exhibited no apparent evidence of oxidation. This is due to the rigid structure of their morphology, which prevented the inner sheets from coming into contact with damp air. Thus, it can be inferred that increasing the lateral dimensions of MXene sheets or producing MXene films by isolating sheets from water using vacuum-assisted filtering may enhance the stability of MXene colloidal solution. (iii) When a liquid solution of MXenes is exposed to air, it becomes very unstable, leading to the oxidation of practically all titanium carbides into TiO_2_. The primary cause of the oxidation of MXenes in an aqueous solution is water, followed by dissolved oxygen. It is essential to identify the primary variables that affect the oxidation of MXenes to design appropriate preventive strategies. Thus, the process of oxidising MXenes may be reduced by optimising storage conditions, such as decreasing the temperature, establishing an atmosphere without oxygen, and filling noble gases like argon. Zhang *et al.*^108^ discovered that by spreading frozen MXenes at low temperatures, they were able to successfully avoid the development of TiO_2_ nanoparticles at the edges of nanosheets. The frozen dispersion of Ti_3_C_2_T_*x*_ has a shape and elemental composition that remains stable for a minimum of 650 days, in contrast to newly synthesised MXenes. On the other hand, the Ti_3_C_2_T_*x*_ dispersion, which was held at room temperature (∼25 °C), exhibited edge erosion within two days. (iv) Materials susceptible to oxidation (such as MXenes) are often treated using physical or chemical methods to provide a protective layer that prevents or blocks redox processes. The primary protection solutions mostly include the grafting of antioxidants onto the surface to create coatings that are resistant to oxidation. Large numbers of visible metal atoms and local positive charges on freshly formed MXenes can be shielded by adding antioxidants or poly-anions with a considerable negative charge. The MXenes' stability will eventually increase with this procedure. Zhang *et al.*^109^ achieved the self-polymerization of dopamine onto the surface of Ti_3_C_2_T_*x*_ nanosheets. The process of freeze-drying and carbonisation was carried out in an inert air atmosphere to create tremella-like MXenes and carbon-layer nano-hybrids. The 2D Ti_3_C_2_T_*x*_ sheets experienced a transformation into a three-dimensional (3D) tremella structure known as T-MXenes@C. This transformation included the application of a thin carbon layer to prevent air oxidation and the aggregation of nanosheets. Besides these MXenes' stability strategies, the stability of MXene-derived TMO-based electrocatalysts is the key factor of this research study. Therefore, the stability of electrocatalysts can be varied through various parameters such as structural prevention, surface tuning, defect engineering, and active site generation.

### 6.2 Transition metal oxides for the OER

**Fig. 9:**
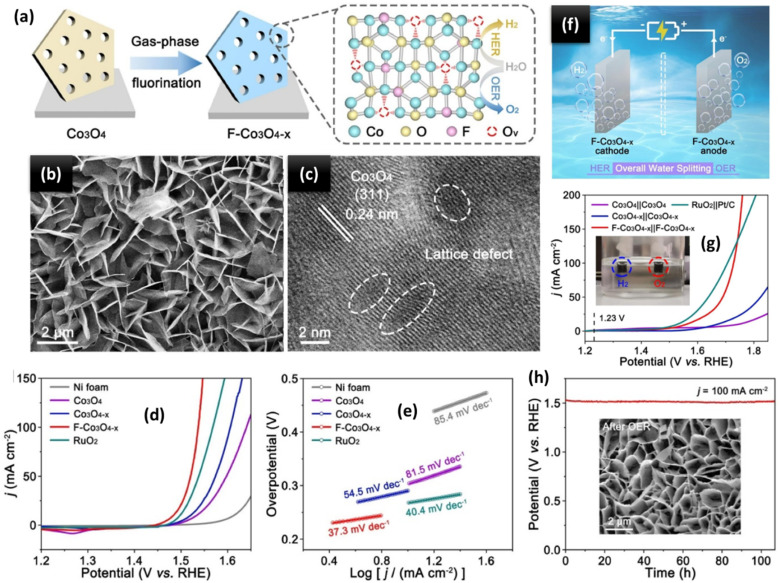
(a) Diagram for the F-Co_3_O_4−*x*_ electrocatalyst's gas-phase fluorination process. (b) SEM of the F-Co_3_O_4−*x*_ electrocatalyst. (c) HR-TEM image of the F-Co_3_O_4−*x*_ electrocatalyst. (d) OER polarisation curves and (e) Tafel plots for the electrocatalysts F-Co_3_O_4−*x*_, Co_3_O_4−*x*_, and Co_3_O_4_. (f) Schematic diagram of an OWS device. (g) OWS polarization curves of the Co_3_O_4_‖Co_3_O_4_, Co_3_O_4−x_‖Co_3_O_4−*x*_ and F-Co_3_O_4−*x*_‖F-Co_3_O_4−*x*_ and commercial RuO_2_‖Pt/C couples, and (h) CP measurements of the F-Co_3_O_4−*x*_ electrocatalyst at 100 mA cm^−2^, with the SEM image displayed in the inset following 100 h of the OER. Reproduced with permission from ref. 117. Copyright 2021, Elsevier.

The exceptional OER activity of TMOs, like perovskite and spinel oxides, has been confirmed due to their affordability, environmental friendliness, ease of synthesis, and relative electrolyte stability.^110^ Moreover, they exhibit variable OER behaviours due to the TMs in their flexible frameworks, which have varying coordination environments and oxidation states. Additionally, doping distinct TMs within a single metal oxide can have a synergistic impact and is a useful strategy for enhancing OER activity. Transition metal hydroxides are one family of lamellar crystal compounds that can even be created *in situ* during the OER process. The catalytic performance can be enhanced by exposing more active sites due to their large specific surface area and the ease with which their composition and electrical characteristics can be adjusted.^111^ Long *et al.*^112^ developed a bifunctional catalyst by growing metallic nickel-decorated TMO nanosheets vertically on a ceria film (ceria/Ni-TMO) and manipulating its composition and surface engineering. When applied as both the cathode and anode in alkaline solutions, the as-synthesized ceria/Ni-TMO showed long-term stability and a low cell voltage of 1.58 V at 10 mA cm^−2^. This was because of the active centres' idealised electronic structure and abundance and the synergistic effect between the *in situ* formed TMO/Ni nanoparticles and the carbon cloth/ceria film. Using Prussian blue analogues (PBAs) as novel metal precursors, Chen *et al.*^113^ synthesized a series of amorphous Co–Fe–V ternary metal oxides with uniformly distributed elements, identical morphologies, and a particularly optimized metal molar composition (denoted as Co_a_Fe_b_V_c_O_*x*_, where *a* + *b* + *c* = 10). Specifically, Co_3_Fe_4_V_3_O_*x*_, whose elemental composition is Co : Fe : V = 3 : 4 : 3, exhibits the highest performance, outperforming a commercial IrO_*x*_ catalyst with an overpotential of only 249 mV to obtain a current density of 10 mA cm^−2^. It also has a low Tafel slope of 41 mV dec^−1^. Qayoom Mugheri *et al.*^114^ investigated the synergistic effects between Co_3_O_4_ and NiO. The resulting composite exhibits promising qualities as a catalyst for alkaline water electrolysis. When compared to its equivalents, the composite material's activity towards the OER increased while its dynamic potential was reduced. Notably, the composite catalyst achieved a low Tafel slope of 101 mV dec^−1^ for the OER. Using two distinct calcination techniques, Zhang *et al.*^115^ prepared pristine Co_3_O_4_ (P-Co_3_O_4_) and Co_3_O_4_-C polyhedrons. A straightforward and environmentally friendly reduction technique was then used to create a reduced Co_3_O_4_ (R-Co_3_O_4_) polyhedron with many surface oxygen vacancies. Doping carbon species and creating oxygen vacancies improved an electrocatalyst's conductivity and electrocatalytic activity. At a current density of 10 mA cm^−2^ in a 1 M KOH solution (pH 13.7), the R-Co_3_O_4_ and Co_3_O_4_-C polyhedrons exhibit lower overpotentials of 380 and 420 mV, respectively, in comparison to the P-Co_3_O_4_ polyhedron (520 mV). Furthermore, their Tafel slopes are 78 and 86 mV dec^−1^, respectively, lower than that of the P-Co_3_O_4_ polyhedron (93 mV dec^−1^). Solangi *et al.*^116^ developed a straightforward hydrothermal growth method for the *in situ* creation of non-stoichiometric CrO_0.87_ and Co_3_O_4_ hybrid materials. CrO_0.87_ was optimised into Co_3_O_4_ nanostructures to better understand its function in the half-cell OER under alkaline conditions. The hybrid material with the largest content of CrO_0.87_ was shown to be particularly effective at driving OER processes with an overpotential of 255 mV at 20 mA cm^−2^. For 45 h, the optimised material showed outstanding endurance with a Tafel slope of 56 mV dec^−1^. Zhang *et al.*^117^ developed a dual-defective Co_3_O_4_ nanoarray (F-Co_3_O_4−*x*_) electrocatalyst with a well-modulated Co-center electronic structure to significantly increase OWS (Fig. 9a). The microscopy results of the F-Co_3_O_4−*x*_ electrocatalyst are depicted (Fig. 9b and c). According to DFT calculations and kinetic studies, F doping and O vacancies can work together to break the water dissociation step's energy barrier limit, which helps alkaline HER produce more hydrogen free radicals (H*). In experiments, F-Co_3_O_4−*x*_ shows very small overpotentials of 77 and 192 mV at 10 and 100 mA cm^−2^, respectively, outperforming the majority of CoO_*x*_-based electrocatalysts that have been reported (Fig. 9d–g). The obtained F-Co_3_O_4−*x*_ as a dual-functional electrocatalyst therefore achieves an ultralow cell voltage of 1.56 V at 10 mA cm^−2^ with a long-term durability of 120 h in an OWS configuration (Fig. 9h). However, at the electrode/electrolyte interface, most TMOs show significant rates of increasing resistance, low ionic diffusivity, and poor electronic conductivity. Introducing a flexible, conductive 2D support such as MXenes on which TMO nanostructures are uniformly placed is an effective way to address these problems. This makes it easier for ions and electrons to move over the interface. Therefore, it is crucial to hybridise MXene and TMO nanostructures in a well-defined architecture in order to integrate the special qualities of the two components in a complementary manner for effective electrochemical activity.

### 6.3 Integrated MXenes and transition metal oxides for the OER

**Fig. 10:**
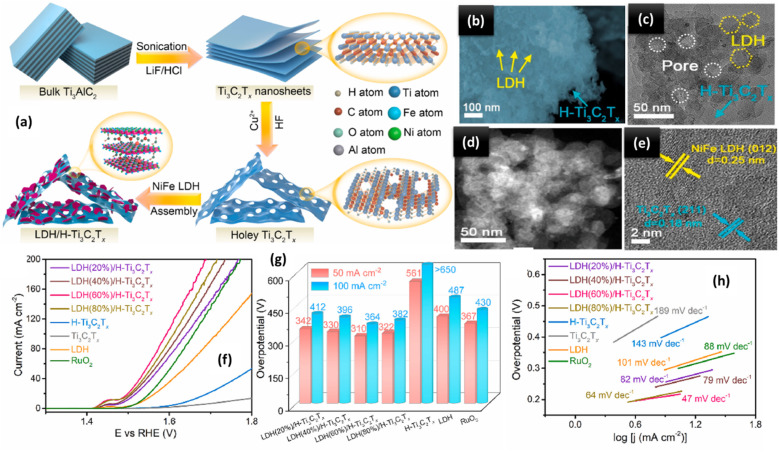
(a) Diagrammatic representation of the LDH/H-Ti_3_C_2_T_*x*_ catalyst's synthesis processes, (b) field emission (FESEM) image, (c) TEM, (d) high-angle annular dark-field imaging and scanning transmission electron microscopy (HAADF-STEM), and (e) HRTEM of the LDH/H-Ti_3_C_2_T_*x*_ nanoarchitecture. The LDH/H-Ti_3_C_2_T_*x*_ nanoarchitecture's OER performance. (f) Representative LSV curves, (g) histograms of required overpotentials and (h) Tafel plots of LDH/H-Ti_3_C_2_T_*x*_ with varying LDH contents, H-Ti_3_C_2_T_*x*_, Ti_3_C_2_T_*x*_, pure LDH and RuO_2_ electrodes in 1 M KOH solution. Reproduced with permission from ref. 118. Copyright 2023, Elsevier.

**Fig. 11:**
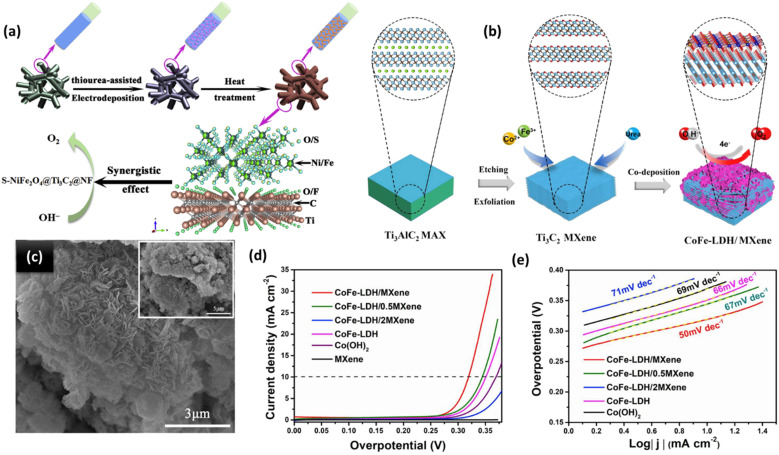
(a) Schematic illustration of the fabrication of the S-NiFe_2_O_4_@Ti_3_C_2_@NF hierarchical network structure. Reproduced with permission from ref. 120. Copyright 2019, Elsevier. (b) Diagram illustrating the method for creating CoFe-LDH/MXene hybrids, (c) SEM image of CoFe-LDH/MXene nanohybrids, (d) LSV curves of samples, and (e) Tafel plots of samples. Reproduced with permission from ref. 121. Copyright 2019, Elsevier.

Integrating MXenes into TMOs might provide an OER hydrophilic, conductive, and active catalyst. Although research on these two materials for the OER is still in its early stages, the MXene incorporated TMO hybrid material appears promising. For instance, Shen *et al.*^118^ showed how to combine *in situ* oxidative etching with a hydrothermal assembly process to produce holey Ti_3_C_2_T_*x*_ MXene nanosheets that are closely linked to ultrathin Ni–Fe LDHs (LDH/H–Ti_3_C_2_T_*x*_) in a controlled manner, confirmed through microscopy results (Fig. 10a–e). As a result, the LDH(60%)/H-Ti_3_C_2_T_*x*_ electrode needs a very low overpotential of only 270 mV to reach 20 mA cm^−2^, which is much better than other LDH/H–Ti_3_C_2_T_*x*_ (277–290 mV) as well as the reference H-Ti_3_C_2_T_*x*_ (464 mV), Ti_3_C_2_T_*x*_ (>650 mV), LDH (336 mV) and RuO_2_ (322 mV) electrodes (Fig. 10f and g). The corresponding Tafel slope of the LDH(60%)/H-Ti_3_C_2_T_*x*_ electrode is calculated to be 47 mV dec^−1^, far surpassing that of LDH (80%)/H-Ti_3_C_2_T_*x*_ (64 mV dec^−1^), LDH (40%)/H-Ti_3_C_2_T_*x*_ (79 mV dec^−1^), LDH (20%)/H-Ti_3_C_2_T_*x*_ (82 mV dec^−1^), RuO_2_ (88 mV dec^−1^), bare LDH (101 mV dec^−1^), and H-Ti_3_C_2_T_*x*_ (143 mV dec^−1^) (Fig. 10h). The cycle stability was also higher than that of both the Ti_3_C_2_T_*x*_ and the bare Ni–Fe LDHs in terms of OER performance. To improve the electrocatalytic performance of the OER, Shinde *et al.*^119^ created novel 2D Ti_3_C_2_ (MXene) sheets coated with NiFe_2_O_4_ nanoparticles. The as-synthesised NiFe_2_O_4_/Ti_3_C_2_ composite demonstrated exceptional kinetic metrics for the electrocatalytic OER, exhibiting a low overpotential of 266 mV at a current density of 10 mA cm^−2^ and a reduced Tafel slope of 73.6 mV dec^−1^. The high metallic conductivity of Ti_3_C_2_ MXene sheets, synergistic effects, and a well-constructed nanoparticle-sheet interface are thought to be the sources of the high electrocatalytic performance of the NiFe_2_O_4_/Ti_3_C_2_ composite. Tang *et al.*^120^ produced absorbed 2D few-layer Ti_3_C_2_ flakes onto a 3D NF network to obtain a three-dimensional (3D) Ti_3_C_2_ based conductive network structure. Following this, ultra-small sulfur-incorporated nickel ferrite nanosheets (S-NiFe_2_O_4_) were produced on the three-dimensional Ti_3_C_2_ based conductive network structure (also known as S-NiFe_2_O_4_@Ti_3_C_2_@NF) by combining a low-temperature calcination technique with a straightforward thiourea-assisted electrodeposition process (Fig. 11a). The as-fabricated NiFe_2_O_4_@Ti_3_C_2_@NF hybrid electrode shows outstanding catalytic stability in 1 M KOH, a Tafel slope of 46.8 mV dec^−1^, and superior OER activity, requiring only 1.50 V *vs.* RHE to achieve a current density of 20 mA cm^−2^. Zaka *et al.*^122^ added TiO_2_ nanoparticles to the surface and layers of V_2_C MXene, which can improve the carbide's capacity for electron transport even further. The as-prepared V_2_C–TiO_2_ nanocomposite electrochemical activity was investigated for OER activity. The composite exhibits an overpotential of 425 mV at a current density of 50 mA cm^−2^. CA was used to assess the electrocatalyst's durability, and the results showed that it was very stable for 48 h with very little variation in current density. Hao *et al.*^121^ synthesized CoFe-LDH (layered double hydroxide) on the surface of Ti_3_C_2_ MXene nanosheets, which demonstrated OER activity than the most advanced RuO_2_ (Fig. 11(b–e)). The improved OER performance may be ascribed to the interaction between the Ti_3_C_2_ MXene substrate's metallic conductivity and CoFe-LDH's oxygen-breaking ability. Ghorbanzadeh *et al.*^123^ developed a CuCo_2_O_4_ nanoparticles/Ti_3_C_2_T_*x*_/NF hybrid electrocatalyst as a stable electrode for the OER, where the electrocatalysts' electrical conductivity may be improved by the inclusion of the Ti_3_C_2_T_*x*_ MXene structure. The results of the experiments suggest that the CuCo_2_O_4_/Ti_3_C_2_T_*x*_ hybrid structure on NF exhibits better OER electrocatalytic activity than pure Ti_3_C_2_T_*x*_. As a result, the hybrid electrocatalyst demonstrated exceptional long-term durability in addition to a low overpotential of 1.67 V at 100 mA cm^−2^ and a lower Tafel slope of 49 mV dec^−1^.

Researchers have also shown that the integration of MXenes with LDHs may be a cutting-edge strategy for applications using renewable energy. Enhanced electrical conductivity and the avoidance of restacking and aggregation are two advantages of coupling LDHs with MXenes. This leads to an increase in the intrinsic activity of the metal in LDH by creating a favourable environment in the electrolyte solution, which in turn results in faster gas kinetics and rapid gas diffusion.^121^ It also generates more active sites. Furthermore, when exposed to the electrolyte, the LDH-MXene structure offers additional metal sites, which boosts the redox reaction's efficiency and speeds it up. For instance, Chen *et al.*^124^ synthesized a hypophosphite-intercalated FeNi (oxy)hydroxide (H_2_PO_2_-/FeNi-LDH-V_2_C) electrocatalyst that works synergistically with V_2_C MXene. With a modest Tafel slope of 46.5 mV dec^−1^ and an overpotential of 250 mV (*η*10) in 1.0 M KOH electrolyte, H_2_PO_2_-/FeNi-LDH-V_2_C demonstrates good OER performance. The composite exhibits notable OER performance and structural stability because of the strong interaction and electrical coupling between FeNi-LDHs and V_2_C MXene, which includes considerable charge transfer. Using a simple *in situ* coprecipitation technique, Wen *et al.*^125^ created a new type of hybrid nanostructure called NiFeCe-LDH/MXene, which was made up of 2D NiFe-layered double hydroxide nanoflakes doped with cerium on the 2D Ti_3_C_2_T_*x*_ MXene surface. Because of the combined effects of MXene coupling and Ce doping, the resulting NiFeCe-LDH/MXene hybrid has a robust interfacial junction, excellent electrical conductivity, and a hierarchical nanoporous structure. Consequently, the hybrid catalyst delivers a low onset overpotential of 197 mV and an overpotential of 260 mV at a current density of 10 mA cm^−2^, demonstrating high catalytic activity for the OER. Using a straightforward hydrothermal technique, Faraji *et al.*^126^ created a heterostructure of NiCoFe-layered double hydroxide (LDH)/Ti_3_C_2_ (MXene)/N-doped carbon nanotube (NCNT). The resulting composite demonstrated excellent electrocatalytic activity towards the OER because of the hybrid porous architecture in NiCoFe-LDH/Ti_3_C_2_ MXene/NCNT, which has a large surface area, high electrical conductivity, lots of active sites, an ideal nitrogen content, and strong electronic interactions. It exhibited a 332 mV overpotential to achieve 10 mA cm^−2^ for the OER. To create hierarchical FeOOH NSs/Ti_3_C_2_, Kaixin Zhao *et al.*^127^ described straightforward ambient growth of FeOOH nanosheets (NSs) on Ti_3_C_2_ nanosheet surfaces. FeOOH and Ti_3_C_2_ have a strong interfacial contact that facilitates rapid charge transfer, enhancing electrocatalytic activity. The hierarchical structure efficiently suppresses the aggregating nanosheets by exposing maximal electro-active surface. In particular, FeOOH NSs/Ti_3_C_2_ exhibits outstanding OER stability with no degradation of current density after 30 h and reaches a current density of 10 mA cm^−2^ at 1.63 V *vs.* RHE with a Tafel slope of 95 mV dec^−1^.

## 7. Summary and outlook for future research

In this comprehensive review, we thoroughly examined the most recent cutting-edge research on the creation of MXene and TMO-based composites for effective OER activity in alkaline media. MXenes have several important functions, including (i) providing a broad electroconductive area and structural support, (ii) acting as an OER catalyst, (iii) stabilising the hybridised material and preventing its aggregation, and (iv) modifying the hybrid's electronic band structure to promote the kinetics of adsorption and desorption. MXene and TMO integrated electrocatalysts strongly inhibit the development of aggregates and clusters. This supports increasing charge transfer and the number of active sites available for electrocatalysis, promoting quick gas diffusion and higher efficiency. There are still some challenges that limit the efficient performance of OER electrocatalysts. The challenges and future perspectives are as follows:

(1) MXene-based electrocatalysts still face certain difficulties when practical water-splitting applications are used. Since HF is a corrosive substance used in most MXene synthesis methods, replacing it in large-scale manufacturing still presents a hurdle. Therefore, the research for integrating MXenes into practical devices and systems has received great attention to determine more scalable synthesis methods for MXenes and MXene-based catalysts suited for large-scale electrocatalytic water splitting applications.

(2) The available information on MXene-based hybrids for OWS only describes the reactions before and after; it does not provide *in situ* characterization, which is necessary to comprehend the catalytic mechanism and catalyst structure evolution during electrocatalytic water-splitting processes. Advanced *in situ* characterization methods should be used to track the surface's evolution, the real catalytically active sites, and the catalytic mechanism.

(3) Agglomeration and restacking of MXene flakes impact the long-term stability and recyclability of MXene-based electrocatalysts. MXenes can be alloyed with different TMOs to avoid this issue. The recent development of MXene and TMO-based OER catalysts has demonstrated significant potential as an effective component for better OER performance. The beneficial effect may be ascribed to the interaction between the active phase and MXene, the increased conductivity due to MXene addition, the improved dispersion of the active phase on the MXene support, and the inhibition of migration and agglomeration because of the immobilisation ability of MXene terminal functional groups.

(4) DFT can be utilized to understand the characteristics of a specific electrode material and identify an ideal structure that would enhance its electrochemical performance. Even with this modest progress, further work is required to resolve unresolved scientific questions and to use these hybrids for real-time energy storage and conversion applications.

## Data availability

No primary research results, software or code have been included and no new data were generated or analysed as part of this review.

## Author contributions

M. Lakhan and A. Hanan: conceptualization, recent progress analysis, writing – reviewing draft, and resources. Y. Wang: writing, reviewing and editing, and methodology. H. Lee: reviewing and editing and methodology. H. Arandiyan: writing, reviewing and editing, project administration, investigation, and supervision.

## Conflicts of interest

The authors declare no competing financial interest.
